# Prevalence and risk factors associated with preterm birth in Ardabil, Iran

**Published:** 2014-01

**Authors:** Rahele Alijahan, Sadegh Hazrati, Mehrdad Mirzarahimi, Farhad Pourfarzi, Peymaneh Ahmadi Hadi

**Affiliations:** 1 *Province Heath Center, Ardabil University of Medical Science, Ardabil, Iran.*; 2 *School of Public Health, Ardabil University of Medical Sciences, Ardabil, Iran.*; 3 *Department of Community Medicine, School of Medicine, Ardabil University of Medical Sciences, Ardabil, Iran.*; 4 *District Health Center, Ardabil University of Medical Science, Ardabil, Iran.*

**Keywords:** *Prevalence*, *Preterm birth*, *Risk factors*

## Abstract

**Background:** Preterm birth is a leading cause of perinatal mortality and long-term morbidity as well as the long-term health consequences and cognitive outcomes.

**Objective: **Present study was conducted to determine prevalence and risk factors associated with preterm birth in Ardabil, Iran.

**Materials and Methods:** A case control study was conducted between Nov 2010 and July 2011 in all three maternal hospitals in Ardabil. All the live newborns during the study period were investigated. Of 6705 live births during the study period 346 births occurred in <37 weeks were taken as a case and 589 term neonates were taken as a control group. Data were obtained through review of prenatal and hospital delivery records. Univariate and multivariate logistic regression analysis were applied to obtain magnitude of association between independent variables and preterm birth.

**Results:** The prevalence rate of preterm birth was 5.1%. History of previous preterm birth (OR=12.7,CI: 3.9-40.4, p<0.001), hypertension (OR=7.3, CI:2.1-25.4, p=0.002), Oligohydramnios (OR=3.9, CI:1.6-9.5, p=0.002), spouse abuse (OR=3.7, CI:1.1-11.8, p=0.024), preeclampsia (OR=3.6, CI:1.3-10.3, p=0.014), premature rupture of membrane (OR=3.1, CI:1.9-4.9, p=0.000), bleeding or spotting during pregnancy (OR=2.0, CI:1.0-3.8, p=0.037), Hyperemesis Gravid arum (OR=2.0, CI: 1.1-3.8, p=0.015), urinary tract infection in 26-30 weeks , (OR=1. 8 CI:1.0-3.2, p=0.04), diastolic blood pressure ≤60 mmg (OR=1.5, CI: 0.99-2.2, p=0.049) were determined as significant risk factors for preterm birth.

**Conclusion:** Early detection and treatment of diseases or disorders among pregnant women especially hypertension, Oligohydramnios, preeclampsia, bleeding or spotting, Hyperemesis Gravid arum, urinary tract infection, and low diastolic blood pressure as well as the improving health care quality delivered to pregnant women may reduce preterm prevalence rate.

## Introduction

Preterm birth, childbirth occurring at less than 37 completed weeks of gestation, is the direct cause for 24% of neonatal deaths (1, 2). Rates of preterm birth have been reported to range from 5-7% of live births in some developed countries and are estimated to be substantially higher in developing countries (1). Comparing with children born at term, preterm infants face to higher risk of several disabilities including neuro-developmental impairments, gastrointestinal complications, cerebral palsy, sensory deficits, learning disabilities, and respiratory illness (3). The morbidity associated with preterm birth often extends to later life resulting in physical, psychological, and economic costs (1, 4). The precise role of events linked to an increased risk of preterm birth is unknown (5). However, there have been a number of previous studies attempting to identify risk factors associated with preterm birth in different countries. In a study conducted in Kerman, Iran; history of preterm labor and history of preterm labor in family were identified as significant risk factors for preterm birth (6).

However, maternal job status, educational level, gravidity, history of periodontal infection, and history of urinary infection have no significant relationships with preterm birth (6). Mahmoodi *et al* found no significant relationships between preterm birth and maternal factors (i.e. age, education, and pre-pregnancy body mass index). However, significant relation was observed between maternal age and premature rupture of membranes (PROM) and PROM was also related to preterm birth significantly (7). Similar study in Yasuj found the risk of preterm labor in women with history of diabetes mellitus, thyroid dysfunction, and cardiac disease to be 2.3 times higher than healthy mothers (8). 

In other studies conducted in Finland , Italy, Brazil, Ireland, and Taiwan risk factors such as smoking, prim parity, anemia, low socioeconomic status, congenital anomalies, hypertension, fetal growth restriction, infection, spontaneous rupture of membranes, multiple pregnancy, cervical dysfunction, antepartum hemorrhage, stress, and malnutrition were associated with the preterm birth (5, 9-12). Estimation of preterm birth rates is required to implement interventions in order to reduce the risk of premature labor and delivery (1). Prevalence of preterm delivery in different cities of Iran is in a range of 5.6-39.4% (13). The etiology of preterm birth is multi factorial and it is affected by social, psychological, biological and genetic factors. Its prevalence also depends on the geographical and demographic features of the population studied and hence results of studies from one area might not be applicable to another (1, 5). This study was carried out to determine the prevalence and risk factors associated with preterm birth in Ardabil, Iran. 

## Materials and methods

All pregnant women hospitalized for delivery in all three maternal hospitals of Ardabil (i.e. Alavi, Sabalan and Arta) were included in a case control study conducted from November 2010 to July 2011. Gestational age was estimated using either the first day of the last normal menstrual period or first trimester obstetric ultrasound for all subjects. Preterm birth, defined as birth occurred in gestational age of less than 37 complete weeks. Termination of pregnancy before 22 weeks of gestation (miscarriage), stillbirth after 22 weeks of gestation, multiple birth, abnormality in uterine, cervical cerclage, women living in other cities, and the women with missing or incomplete prenatal care records were excluded from the study. Including criteria for cases were defined as singleton live births occurred in less than 37 complete weeks. Control group considered as the singleton live births occurred at 37 or more completed weeks of gestation. 

Of 6705 live births during the study period 346 births occurred at <37 completed weeks and they were considered as cases. Control group (N=589) was randomly selected from singleton live births occurred at 37 or more completed weeks of gestation. In order to improve the accuracy and compensate of possible sample loss, the number of newborns in the control group was approximately twice the cases. That is, per every birth occurred at <37 completed weeks, if possible the next two newborns with gestational age of 37 or more completed weeks were selected as control. Data were collected retrospectively through review of prenatal and hospital delivery records using a self-designed questionnaire. 

The questionnaire contained socio-demographic factors and maternal and prenatal care characteristics of current and previous pregnancies. Socio-demographic factors included: age, education level, social class, maternal occupation, parents' consanguinity, and area of residence (rural or urban). Maternal and prenatal care characteristics in current pregnancy covered body mass index, maternal height, maternal weight gain after 15 weeks of gestation, gestational diabetes, positive Hepatitis B surface antibody (HbsAb) antigen, hypertension in pregnancy, low systolic blood pressure (≤100), low diastolic blood pressure (≤60), hyperemesis gravid arum, unwanted pregnancy, pre-pregnancy care, urinary tract infection during pregnancy in 6-10 weeks, urinary tract infection during pregnancy in 26-30 weeks, bleeding or spotting, spouse abuse, ferrous and multivitamin use, preterm rupture of membrane, hospitalization in pregnancy, dental problems, chronic diseases in pregnancy, location of placenta, amniotic fluid volume, taking medicine during pregnancy, hard working in pregnancy, infertility history and treatment, mode of delivery, congenital abnormalities, previous contraceptive method, hemoglobin level less than 11g/d in 6-10 weeks of pregnancy, hemoglobin level less than 10.5 g/d in 26-30 weeks of pregnancy, smoking, opium and alcohol consumption.

Previous pregnancy and reproductive history characteristics included; gravidity, parity, history of abortion, number of previous abortion, interval from previous abortion, birth interval, history of still birth, history of preterm labor, history of neonatal congenital abnormalities, history of term Low Birth Weight infant, and previous caesarian delivery. The term preeclampsia was used for woman with hypertension (i.e. blood pressure ≥140/90 mmHg) and proteinuria (i.e. ≥300 mg in a 24-hour sample or ≥30 mg/dl in a single sample). 

Hypertension before pregnancy or experience of hypertension in early stages of pregnancy (before 20 weeks) without proteinuria referred to chronic high blood pressure (14). Urinary tract infection is diagnosed where asymptomatic bacteruria was present on positive urine culture test, spouse abuse included emotional abuse or physical violence, and emotional abuse was defined as repeated yelling, humiliating, and threatening acts by the male partner during pregnancy (14, 15). Physical abuse was considered as one or more acts of physical aggression perpetrated by the male partner during pregnancy, premature rupture of membrane or leakage of amniotic fluid referred to a rupture that occurred before the onset of regular contraction or before 37 weeks of pregnancy (14, 16). 

Treatment of infertility referred to the current pregnancy that was the result of infertility treatment methods and maternal chronic diseases were referred to cardiovascular, hepatic, renal, and lung diseases (17). Hypotension was considered as blood pressure equal or less than100/60 mm/Hg (18). We also defined hypermetric pregnancies as those vomiting in the pregnancy period at a such severity as to require the patient admission to hospital (19). This project (code, 893056) was approved by ARUMS medical research ethics committee and subjects got consent for participation. 

**Statistical analysis**

Chi-square, univariate and multivariate logistic regression were applied to analyze data using SPSS version 16. Chi-square was employed to compare distribution of variables between the groups, univariate analysis was applied to evaluate associations between independent variables and preterm birth. In order to highlight important risk factors for preterm birth, multivariate regression analysis was conducted and all the variables displaying significant relationships with preterm birth in the univariate analysis were entered into the model (Forward Wald Model). For the logistic regression, results are reported as odds ratios and 95% confidence intervals (CI) along with P values. SPSS version 16 was used to conduct these statistical analyses. 

## Results

Out of 6705 live newborns delivered, 346 (5.1%) were preterm. Among the variables considered as socioeconomic characteristics, only social class of parents was significantly correlated with preterm delivery (OR= 1.4, CI: 1.0-1.8, p=0.016). The results for statistical analysis of maternal and prenatal care characteristics of the subjects in current pregnancy in relation with preterm birth are summarized in table I. 

The odds of delivering a preterm birth was significantly high among the women with short stature, gestational diabetes, pre-eclampsia, hypertension, positive HBS antigen, diastolic hypotension, hyperemesis gravid arum, urinary tract infection in 6-10 weeks and 26-30 weeks of gestation, spotting or bleeding, suffering from spouse abuse, preterm rupture of membrane, hospitalization during pregnancy, chronic disease, Oligohydramnios, taking medicine during pregnancy, and women involved in heavy physical works. Comparing to control group, although the differences were not statistically significant, we observed higher prevalence rates of preterm birth in women with; BMI 19/8> Kg/m2, weight gain of less than 2500 g/ week after the 15^th^ week of pregnancy, low systolic blood pressure, polyhydramnion, pregnancy through In vitro fertilization (IVF), pregnancy through ovular stimulator drugs consumption and congenital abnormalities (Table I). 

Other variables studied associated with no increase in the rate of preterm births. Smoking, alcohol consumption, and opiate use were extremely rare and that they were excluded from statistical analyses. The results obtained for univariate analysis of preterm deliveries in relation with previous pregnancy and reproductive characteristics of the subjects are shown in table II. Women with previous experience of abortion, a birth interval of less than 3 years, history of preterm birth, and women with history of low birth weight delivery significantly were more likely to deliver preterm infant. We observed incidence rates of preterm birth to be higher in women with three or more parity, experiencing three or more previous abortions, history of still birth, and history of delivering term low birth weight, however, the differences were not statistically significant (Table II). Based on the results obtained from multivariate analysis; history of preterm birth, hypertension during pregnancy, oligohydramnios, spouse abuse, preeclampsia, premature rupture of membrane, bleeding or spotting during pregnancy, hyperemesis gravid arum, urinary tract infection in 26-30 weeks of pregnancy, middle and low social class, and diastolic hypotension were identified as significant risk factors for preterm birth delivery (Table III).

**Table I:** Univariate analysis of maternal and prenatal care characteristics in current pregnancy

		**Preterm n (%)**	**Term n (%)**	**Total n (%)**	**OR (95%CI)**	**p-value**
Body mass index						
	19/8>	39 (12.5)	51 (9.3)	90 (10.4)	1.27 (0.80-2.01)	0.305
	19/8-26	158 (50.5)	263 (47.8)	421 (48.8)	1 (ref)	ref
	26/1-29/9	67 (21.4)	143 (26.0)	210 (24.3)	0.78 (0.54-1.10)	0.165
	30≤	49 (15.7)	93 (16.9)	142 (16.5)	0.87 (058-1.30)	0.518
Maternal height						
	155>	101 (30.9)	140 (24.7)	241 (27.0)	1.36 (1.00-1.84)	0.046
	155≤	226 (69.1)	426 (75.3)	652 (63.0)	1 (ref)	
Maternal weight gain after 15 weeks of gestation.						
	<2500 gr/week	12 (3.9)	10 (1.8)	22 (2.5)	2.20 (0.94-5.10)	0.06
	2500≤ gr/ week	298 (96.1)	547 (98.2)	845 (97.5)	1 (ref)	
Gestational diabetes						
	Yes	15 (4.4)	11 (1.9)	26 (2.8)	2.41 (1.09-5.32)	0.029
	No	323 (95.6)	572 (98.1)	895 (97.2)	1 (ref)	
Positive HBS antigen						
	Yes	6 (2.6)	2 (0.5)	8 (1.2)	5.10 (1.14-28.50)	0.035
	No	229 (97.4)	436 (99.5)	665 (98.8)	1 (ref)	
Hypertension in pregnancy						
	Hypertension	17 (5.0)	8 (1.4)	25 (2.7)	4.0 (1.71-9.40)	0.001
	Preeclampsia	28 (8.2)	12 (2.0)	40 (4.3)	4.40 (2.20-8.81)	0.000
	No hypertension	298 (86.9)	566 (96.6)	864 (93.0)	1 (ref)	Ref
Low systolic blood pressure (100≥)						
	Yes	154 (46.7)	243 (42.9)	397 (44.3)	1.16 (0.88-1.53)	0.267
	No	176 (53.3)	324 (57.1)	500 (55.7)	1 (ref)	
Low diastolic blood pressure (60≥)						
	Yes	137 (41.5)	197 (34.7)	334 (37.2)	1.33 (1.00-1.76)	0.043
	No	193 (58.5)	370 (65.3)	563 (62.8)	1 (ref)	
Hyperemesis gravidarum						
	Yes	57 (16.6)	62 (10.5)	119 (12.9)	1.69 (1.14-2.49)	0.008
	No	286 (83.4)	526 (89.5)	812 (87.2)	1 (ref)	
Urinary tract infection during pregnancy in 6-10week						
	Yes	61 (18.4)	71 (12.5)	132 (14.7)	1.57 (1.08-2.29)	0.016
	No	271 (81.6)	498 (87.5)	769 (85.3)	1 (ref)	
Urinary tract infection during pregnancy in 26-30week						
	Yes	37 (13.9)	45 (8.9)	82 (10.6)	1.65 (1.04-2.63)	0.033
	No	229 (86.1)	461 (91.1)	690 (89.4)	1 (ref)	
Bleeding or spotting						
	Yes	55 (16.3)	39 (6.7)	94 (10.2)	2.71 (1.75-4.19)	0.000
	No	283 (83.7)	545 (93.3)	828 (89.8)	1 (ref)	
Spouse abuse						
	Yes	19 (5.6)	8 (1.4)	27 (2.9)	4.18 (1.81-9.67)	0.001
	No	322 (94.4)	568 (98.6)	890 (97.1)	1 (ref)	
Preterm rupture of membrane						
	Yes	95 (28.7)	96 (16.6)	191 (21.0)	2.02 (1.46-2.79)	0.000
	No	236 (71.3)	483 (83.4)	719 (79.0)	1 (ref)	
Hospitalization in pregnancy						
	Yes	61 (18.2)	54 (9.5)	115 (12.7)	2.11 (1.42-3.13)	0.000
	No	275 (81.8)	514 (90.5)	789 (87.3)	1 (ref)	
Dental problems						
	Yes	136 (43.3)	267 (49.4)	403 (47.1)	0.78 (0.593-1.03)	0.088
	No	178 (56.7)	274 (50.6)	452 (52.9)	1 (ref)	
Chronic disease in pregnancy						
	Yes	25 (7.3)	15 (2.6)	40 (4.3)	2.98 (1.55-5.74)	0.001
	No	319 (92.7)	571 (97.4)	890 (95.7)	1 (ref)	
Location of placenta						
	Anterior	132 (46.5)	227 (43.7)	359 (44.7)	1.04 (0.710-1.55)	0.811
	Posterior	88 (31.0)	164 (31.5)	252 (31.3)	0.96 (0.63-1.46)	0.878
	Fundul	56 (19.7)	101 (19.4)	157 (19.5)	1 (ref)	ref
	Lateral	8 (2.8)	28 (5.4)	36 (4.5)	0.51 (0.22-1.30)	0.127
Amniotic fluid volume						
	Normal	263 (86.5)	514 (95.4)	777 (92.2)	1 (ref)	ref
	Oligohydramnios	33 (10.3)	12 (2.2)	45 (5.3)	5.3 (2.73-10.50)	0.000
	Polyhydramnion	8 (2.6)	13 (2.4)	21 (2.5)	1.20 (0.49-2.93)	0.685
Taking medicine in pregnancy						
	Yes	37 (10.9)	32 (5.5)	69 (7.5)	2.09 (1.27-3.43)	0.003
	No	302 (89.1)	547 (94.5)	849 (92.5)	1 (ref)	
Hard working in pregnancy						
	Yes	26 (7.7)	24 (4.2)	50 (5.5)	1.90 (1.07-3.38)	0.027
	No	310 (92.3)	546 (95.8)	586 (94.5)	1 (ref)	
History of Infertility and treatment method						
	IUI	1 (0.3)	2 (0.3)	3 (0.3)	0.86 (0.07-9.57)	0.906
	IVF	3 (0.9)	1 (0.2)	4 (0.4)	5.1 (0.53-5.13)	0.155
	Ovular stimulator drugs	11 (3.2)	12 (2.0)	23 (2.5)	1.58 (0.69-3.63)	0.276
	Non Treated infertile women	1 (0.3)	3 (0.5)	4 (0.4)	0.57 (0.06-5.56)	0.633
	Non infertile women	330 (95.4)	571 (96.9)	902 (96.4)	1 (ref)	ref
Mode of delivery						
	Normal delivery	135 (39.0)	194 (32.9)	329 (35.2)	1.30 (0.98-1.71)	0.060
	Caesarian delivery	211 (61.0)	395 (67.1)	606 (64.8)	1 (ref)	
Congenital abnormalities						
	Yes	18 (5.2)	27 (4.6)	45 (4.8)	1.14 (0.620-2.10)	0.670
	No	328 (94.8)	562 (95.4)	890 (95.2)	1 (ref)	
						

* was considered as reference group in statistical analysis.

**Table II:** Univariate analysis of previous pregnancy and reproductive characteristics

		**Preterm n (%)**	**Term n (%)**	**Total n (%)**	**OR (95%CI)**	**p-value**
Gravidity						
	Prim gravid	158 (46.1)	279 (47.6)	437 (47.0)	1 (ref)	0.649
	Multi gravid	185 (53.9)	307 (52.4)	492 (53.0)	1.0 (0.81-1.39)	
Parity						
	O	178 (51.7)	297 (50.7)	475 (51.1)	1 (ref)	
	1	103 (29.9)	195 (33.3)	298 (32.0)	0.88 (0.65-1.19)	0.413
	2	45 (13.1)	72 (12.3)	117 (12.6)	1.0 (0.68-1.58)	0.843
	3≤	18 (5.2)	22 (3.8)	40 (4.3)	1.36 (0.71-3.61)	0.848
History of abortion						
	Yes	69 (20.0)	80 (13.6)	149 (16.0)	1.58 (1.11-2.25)	0.011
	No	276 (80.0)	507 (86.4)	783 (84.0)	1 (ref)	
Number of previous abortion						
	3>	337 (98.0)	581 (99.3)	918 (98.8)	1 (ref)	0.08
	3≤	7 (2.0)	4 (0.7)	11 (1.2)	3.0 (0.87-10.38)	
Interval from previous abortion						
	1>	23 (35.9)	27 (38.0)	50 (37.0)	0.73 (0.29-1.86)	0.522
	1-3	26 (40.6)	31 (43.7)	57 (42.2)	0.72 (0.29-1.81)	0.491
	>3	15 (23.4)	13 (18.3)	28 (20.7)	1 (ref)	
Birth interval						
	>1	8 (5.4)	4 (1.4)	14 (2.8)	4.50 (1.33-15.4)	0.016
	1-3	48 (32.2)	63 (22.7)	111 (26.0)	1.7 (1.10-2.70)	0.017
	>3	93 (62.4)	211 (75.9)	304 (71.2)	1 (ref)	
History of still birth						
	Yes	9 (2.6)	10 (1.7)	19 (2.0)	1.54 (0.620-3.83)	0.351
	No	335 (97.4)	574 (98.3)	909 (98.0)	1 (ref)	
History of preterm labor						
	Yes	25 (7.2)	7 (1.2)	32 (3.4)	6.4 (2.75-15.0)	0.000
	No	320 (92.8)	577 (98.8)	897 (96.6)	1 (ref)	
History of neonatal congenital abnormalities						
	Yes	5 (1.5)	9 (1.5)	14 (1.5)	0.94 (0.33-2.83)	0.913
	No	338 (98.5)	572 (98.5)	910 (98.5)	1 (ref)	
History of term low birth weight infant						
	Yes	8 (2.3)	6 (1.0)	14 (1.5)	2.2 (0.78-6.64)	0.129
	No	336 (97.7)	576 (99.0)	912 (98.5)	1 (ref)	
Previous caesarian delivery						
	Yes	81 (23.5)	136 (23.3)	217 (23.4)	1.0 (0.73-1.38)	0.958
	No	264 (76.5)	447 (76.7)	711 (76.6)	1 (ref)	

**Table III:** Multivariate logistic regression analysis of risk factors associated with preterm birth

**Risk factors**	**Preterm**			**Term**
	**OR**	**CI**	**p-value**	
History of preterm birth	12.7.	3.9-40.4,	0.000	1
Hypertension during pregnancy	7.3,	2.1-25.4	0.002	1
Oligohydramnios	3.9	1.6-9.5	0.002	1
Spouse abuse	3.7	1.1-11.8	0.024	1
Preeclampsia	3.6	1.3-10.3	0.014	1
Premature rupture of membrane	3.1	1.9-4.9	0.000	1
Bleeding or spotting during pregnancy	2.0	1.0-3.8	0.037	1
Hyperemesis Gravid arum	2.0	1.1-3.8	0.015	1
Urinary tract infection in 26-30weeks of pregnancy	1.8	1.0-3.2	0.044	1
Middle and low social class	1.6	1.0-2.3	0.021	1
Diastolic blood pressure ≤60 mmg (Diastolic Hypotension)	1.5	0.99-2.2	0.049	1

## Discussion

The prevalence of preterm birth found in current study (i.e. 5.1%) is appreciably lower than the rates reported for African (12.6%), Asian (9.8%), and some European countries (6.7%) as well as the figures reported for Vietnam (i.e. 11.8%) but is higher than rate of 4.4% reported for Italy (1, 5, 20). However, the rate of preterm birth found in present study is similar to those of studies conducted in Islamic Republic of Iran (i.e. Qom 5.6% and Mashhad 6.1%) (13). The low rate of preterm birth found in present study might reflect success of different programs introduced by Iranian Health Ministry to improve health service quality delivered to pregnant women including pre-pregnancy and pregnancy health care in the last decade.

Experience of previous preterm birth was identified as the most significant risk factor for preterm birth with odds ratio of 12.7. This is in line with the findings of studies where women with previous preterm delivery were at increased risk for their next pregnancy (5, 6, 20, 21). The recurrence risk in women with a previous preterm delivery ranges from 15% to more than 50% depending on the number and gestational age of previous deliveries (4). The mechanism for this has not been well understood, however, the likelihood of such experience among the women with prior spontaneous labor as well as those with inducing preterm birth is rising. 

Persisting or recurrent intrauterine infection during several pregnancies along with the disorders associated with preterm birth (e.g. gestational diabetes, hypertension, and obesity) that tend to last from one pregnancy to the next, might explain many repetitive spontaneous and induced preterm births (4). Other important risk factors were hypertension and pre-eclampsia that increased the risk of preterm birth by 7.3 and 3.6 folds, respectively. 

Hypertension increases resistance of uterine vessels and reduce uteroplacental fluid, which in turn causes intrauterine growth restriction (22). Moreover, the high rate of disorders like placenta abruption and pre-eclampsia and intrauterine growth restriction among women with hypertension may results in surgical operations and preterm birth (14). Although the difference was not statistically significant; Renzo *et al* reported the likelihood of preterm birth to be 2.6 times greater among women with chronic hypertension (5). Various factors including fetal abnormalities, hypertension, pre-eclampsia, blood transfusion between twins, and chronic leakage of ammonite in ruptured areas of the membrane may lead to Oligohydramnios (14). Some reports have estimated the likelihood of preterm birth to be 3-10 times higher in women with Oligohydramnios (12, 14). 

This figure for present study was 3.9. preterm rupture of membrane is the most common cause of Oligohydramnios (14). Similar to present study preterm rupture of membrane has been reported to be related significantly to preterm birth (7). Odds ratio of preterm birth in women experiencing physical and emotional abuse was 3.4 that agrees well with previous report where an odds ratio of 2.4 was observed for such women (23). Researchers investigating this issue found inconsistent results. Rodrigues *et al* reported that 24% of the women experiencing spouse abuse and 8% of the women without history of such abuse had preterm birth (24). 

Similarly, another study found these figures to be 5.3% among women experiencing violence and 1.2% within those without such experiences (25). However, other studies have reported no significant relationship between domestic violence and preterm birth (26-28). Women experiencing spouse abuse may also have conditions such as sexually transmitted diseases, vaginal bleeding, depression, and stress that increase level of inflammatory factors and consequently leading to preterm birth (4,    16 ). In addition, physical harassment may result in injuries of placenta, preterm rupture of the membrane or the release of prostaglandins (16).

Hyperemesis gravid arum occurs in approximately 0.3-2% of pregnancies (29). Some studies have not found hyperemesis to be a risk factor for preterm delivery (19, 30). However, similar to our findings, others reported that the risk of prematurity to increase 1.3 times in women who suffered from hyperemesis gravid arum (31, 32). Vaginal bleeding in pregnancy increased the odds of delivering immature babies up to 2.2 times, which is in agreement with the findings where the risk of preterm birth was higher in women with bleeding or spotting during their pregnancy (33, 34). The reason for vaginal bleeding during the first half of pregnancy is unknown in most cases. However, those with bleeding in second half of pregnancy have placenta abruption or placenta previa (22). Vaginal bleeding as an indication of serious consequences of pregnancy may cause fetal or maternal emergencies leading to induced preterm birth. 

The infection of the urinary system is the most prevalent bacterial infections occurred during pregnancy (14). Similar to our results, Schieve *et al* has considered urinary system infection as a risk factor for premature birth (35). Infection may raise release of inflammatory chemokine’s and cytokines such as interleukins and tumor necrosis factors. Microbial Endotoxins and proinflammatory cytokines stimulate the production of prostaglandins (other inflammatory mediators) and matrix-degrading enzymes that finally result in stimulation of uterine contractions, preterm rupture of the membrane, and preterm birth (14). 

However, some studies found no significant correlations between preterm birth and urinary infection (6, 35-37). Similar to recent findings low socioeconomic status was found to be an important factor affecting preterm deliveries (9, 20, 21). This might be attributed to the fact that low income women normally suffer from nutritional deficiency, insufficient health care, low education, drug abuse, cigarettes and alcohol consumption, domestic violence, and stressful life (11, 22). However, a study performed in 1997 found no association between socioeconomic status and preterm birth (38). 

Maternal hypotension during pregnancy (often defined as blood pressure ≤110/60), may be associated with reduced utero-placental perfusion, prematurity, and low birth weight (39). Studies characterizing relationships between maternal hypotension and prematurity have reported inconsistent results (18, 39, 40). In our study maternal diastolic hypotension increased the risk of preterm delivery by a factor of 1.5. Treatment of hypertensive pregnant women may improve the placental perfusion and fetal outcomes (41).

The data for some variables were collected based on the medical records of the subjects and that; there might be a potential risk for quality of recorded data. Other limitation was that, where the mothers did not remember the date of the first day of last menstrual period accurately, we calculated gestational age by obstetric ultrasound taken in first trimester. To insure the accuracy of data the obstetric ultrasound should be performed by the same person and the same machine for all participants; while it was not possible in our study. 

## Conclusion

History of preterm birth, hypertension and pre-eclampsia, preterm rupture of the membrane, oligohydramnios, spotting and bleeding, urinary infection, hyperemesis gravid arum, and low social status and low diastolic blood pressure were identified as the most important risk factors for preterm birth. Identifying pregnant women at the risk of preterm delivery and proving quality healthcare may decrease the rate of preterm birth and its consequences.
